# Salivary Microbiome Profiling of HPV+ and HPV− Oropharyngeal Head and Neck Cancer Patients Undergoing Durvalumab Immunotherapy Suggests *Prevotella melaninogenica* and *Veillonella atypica* as Key Players: A Pilot Study

**DOI:** 10.3390/cancers17030452

**Published:** 2025-01-28

**Authors:** Jean-Luc Mougeot, Micaela Beckman, Mitra Kooshki, Justin Neuberger, Kirtikar Shukla, Cristina Furdui, Farah Bahrani Mougeot, Mercedes Porosnicu

**Affiliations:** 1 Carolinas Medical Center, Atrium Health, Charlotte, NC 28203, USA; jean-luc.mougeot@atriumhealth.org (J.-L.M.); micaela.beckman@atriumhealth.org (M.B.); 2 School of Medicine, Wake Forest University, Winston-Salem, NC 27109, USA; mkooshki@wakehealth.edu (M.K.); justin.neuberger@atriumhealth.org (J.N.); kshukla@wakehealth.edu (K.S.); cfurdui@wakehealth.edu (C.F.)

**Keywords:** head and neck cancer, HPV, immunotherapy, durvalumab, salivary microbiome

## Abstract

Objective: Head and neck cancer (HNC) is a common cancer represented by nearly 80% oral cavity (OC) and oropharyngeal cancers (OPCs). Seventy percent of OPCs are associated with the Human Papilloma Virus (HPV). Immunotherapy holds the promise of future improvements in treating HNC patients. The study objective was to determine whether durvalumab immunotherapy alone, prior to curative surgery, would significantly impact the oral salivary microbiome in a pilot cohort of HPV negative and positive OC and OPC patients. Methods: Early stage OPC patients with squamous cell carcinoma were recruited: 5 HPV+ and 12 HPV−, and treated with two or three administrations of durvalumab given every two weeks, prior to surgery. Unstimulated saliva was collected and processed for bacterial DNA Isolation and V1–V3 16S rRNA gene next generation sequencing, taxa identification, and determination of relative abundance at four time points: baseline prior to surgery (A) and weekly durvalumab treatment timepoints (B, C, and D). Alpha- and beta-diversity differences for the time series were determined in Primer_v7_. MaAsLin2 in R was used to identify potential associations with the time series and/or HPV status. Linear decomposition model (LDM) R-package was used to investigate the relationship of salivary microbiome with HPV status. ROC curves were plotted for significant species in common between MaAsLin2 analysis and FDR-corrected Mann-Whitney U-test using XLSTAT. Results: Longitudinal microbiome data across four timepoints (A, B, C, D) were obtained (HPV+: n = 18 samples; HPV−: n = 46 samples). A total of 416 taxa were detected across all time points, ranging from 336 to 373 per group. There were no differences in *α*- and *β*-diversities for all longitudinal comparisons (C vs. BCD, AB vs. CD, or A vs. B, C, or D). However, comparison A vs. D showed a significant increase in *Prevotella melaninogenica* relative abundance, a potentially pathogenic species able to evade the immune system, after three weeks treatment. Moreover, differences in *beta*-diversity based on HPV status were found. LDM analysis identified *Veillonella atypica*, overrepresented in HPV+ group, as the top species accounting for HPV status. Conclusions: The results are consistent with findings from previous studies investigating HNC patients treated with chemoradiotherapy. More research is needed to understand possible impact of immunotherapy on opportunistic bacterial species, although negligible impact from durvalumab treatment on salivary microbiome was observed.

## Simple Summary

Head and neck cancer (HNC) affects about 67,000 people each year, representing 3.4% of cancers in the United States. With the advent of novel immunotherapies, little is known about their potential effects on the oral microbiome. Perturbation of the oral microbiome has been associated with oral complications following cancer treatment such as high dose chemotherapy and radiation therapy. Our objective was to determine whether durvalumab immunotherapy would have major effects on the oral microbiome. Our findings show that it is not the case. However, our results also suggest that opportunistic oral bacterial pathogens could evade the immune system, which would warrant monitoring of HNC patients during or after immunotherapy treatment.

## 1. Introduction

Head and neck squamous cell carcinomas account for nearly 5% of all cancer diagnoses and deaths per 2020 GLOBOCAN estimates [1]. Risk factors for head and neck cancer (HNC) include tobacco use, alcohol consumption, human papillomavirus (HPV) infection, and/or Epstein–Barr virus (EBV) infection [2]. Specifically, HPV infection accounts for more than 70% of oropharynx squamous cell carcinomas in developed nations [2].

There are over 170 different types of HPV able to infect stratified epithelium, with HPV16 being responsible for up to 90% of HPV-related oropharyngeal HNSCC cases in North America [2,3]. Two HPV oncogenes, E6 and E7, promote malignancy through anti-apoptotic effects and mediate epidermal growth factor receptor-regulated cell proliferation [4,5]. While E6 promotes tumor suppressor TP53 degradation, E7 can inhibit tumor suppressor RB Transcriptional Corepressor 1 protein by direct binding, thereby promoting cell cycle progression [4,6].

Little is known about possible direct or indirect cooperative associations between the oral microbiome and HPV infection prior to cancer development, once HNC has established, or during cancer treatment. Recent studies have investigated HPV infection and the role of oral microbiome in HNC patients [7,8,9,10,11].

A review by Shigeishi et al. suggested that the oral species, *Treponema denticola* and *Fusobacterium nucleatum*, previously linked to periodontal disease, were associated with HPV16 positivity in gingival crevicular fluid [12,13]. Periodontal disease perturbs the normal oral microbiota leading to dysbiosis, which translates into more prevalent higher relative abundance of opportunistic pathogens such as *T. denticola*, *F. nucleatum*, and *Porphyromonas gingivalis* in the oral cavity. These species can produce virulence factors resulting in proinflammatory cytokines from squamous cells that lead to inflammation [14]. Patients with periodontitis have been reported to have a 2.63-fold increased risk for HNC [15].

Previously, our group found *Haemophilus pittmaniae* and *Leptotrichia* spp. to be part of a multi-marker taxa signature HPV positive HNC patients using samples from multiple sites in oral cavity [11]. Our results were in line with a previous study that had shown a higher prevalence of *Leptotrichia amnionii* in women with cervical cancer (38.5%) compared to women without cancer (11.1%) [16].

Cancer treatment such as chemoradiation therapy alters the composition of the oral microbiome, influencing oral complications [17,18]. However, little is known about the effects of immunotherapy on the oral microbiome. In this study, we performed bacterial 16S rRNA gene sequencing of saliva samples to determine the effects of durvalumab immunotherapy alone, as first line treatment before curative surgery, and on oral microbiome profiles in patients with head and neck squamous cell carcinoma (HNSCC). The saliva microbiome profiles were assessed at baseline and then at one-week intervals through treatment, up to three weeks.

## 2. Methods

### 2.1. Patient Recruitment and Treatment

Patients were recruited according to an investigated initiated pilot clinical study approved by the Wake Forest Comprehensive Cancer Center Institutional Review Board (IRB00038877; approval date 20 May 2016) and registered with clinicalstudies.gov (NCT 02827838). The study aimed to investigate the differential clinical and tissue effect of a short course of the PD-L1 inhibitor durvalumab in previously untreated patients with HPV positive and negative HNSCC. Patients with early stage operable HNSCC of the OC and OP who were scheduled for curative intent surgery were recruited and treated with two or three administrations of durvalumab 750 mg every two weeks until the time of surgery. HPV status was checked by PCR in patients with oropharyngeal cancer. The pilot patient cohort consisted of five HPV positive (HPV+) and 12 HPV negative (HPV−) patients. Biopsy samples at diagnosis and pathology samples from the surgical specimen, as well as weekly blood and saliva samples, were collected, processed, and stored at −80 °C in a biospecimen repository.

### 2.2. Saliva Sample Collection

Unstimulated whole saliva was collected by passive drool technique, with a targeted volume of 5 cc per sample. Patients were instructed to not brush their teeth within 45 min prior to sample collection and to not have any dental work within 24 h prior to sample collection. Patients were instructed to allow saliva to pool in the mouth and, with head tilted forward, to allow the saliva to drool into the tube. Once the sample was collected, the tube was placed in an ice box and transported immediately to the lab, where it was processed immediately by centrifugation at 2600× *g* for 15 min at 4 °C. Pellets were stored at −80 °C until further use for microbial DNA analysis.

### 2.3. Bacterial DNA Isolation and 16S Bacterial Taxa Identification

Bacterial genomic DNA was extracted from frozen saliva pellets using the ZymoBIOMICS-96 MagBead DNA kit (Zymo Research, Irvine, CA). Bacterial 16S rRNA gene next generation sequencing (NGS) was performed using the Quick-16S NGS Library Prep Kit amplifying the V3–V4 gene region with a reported coverage rate of ~84%. Publicly available 16S databases (e.g., Greengenes, SILVA, etc.) indicate a coverage between 65% and 83% at the species level, confirming that the Zymo database provided satisfactory V3–V4 taxonomy assignment. Library preparation was performed using real-time PCR to control cycle threshold and limit PCR chimera formation. Final PCR products were quantified using qPCR fluorescence readings and pooled based on equal molarity. The final pooled library was cleaned with the Select-a-Size DNA Clean & Concentrator kit (Zymo Research, Irvine, CA, USA) and quantified using TapeStation (Agilent Technologies, Santa Clara, CA, USA) and Qubit (Thermo Fisher Scientific, Waltham, WA, USA). The ZymoBIOMICS Microbial Community Standard (Zymo Research, Irvine, CA, USA) was used as a positive control for each DNA extraction and library preparation. Negative controls were used to assess the level of bioburden by the wet-lab process. Sequencing was completed using the Illumina Nextseq (Illumina, Inc., Hayward, CA, USA) with P1 reagent kit (600 cycles) performed with 30% PhiX spike-in. Unique amplicon sequence variants were inferred from raw reads using the Dada2 pipeline, as previously described [19]. Sequencing errors and chimeric sequences were removed with the Dada2 pipeline. Taxonomy assignment was performed using Uclust from Qiime_v1_._9_._1_ with a 16S Zymo database (Zymo Research, Irvine, CA, USA). Dada2 and Uclust parameter settings are available on https://github.com/mbeckm01/HPV_OSCC (accessed on 20 December 2024) (Supplementary File S1). Publicly available 16S databases were avoided due to possibility of containing errors, inconsistencies, and poor-quality sequences, whereas the 16S Zymo database is continuously monitored with simulated controls to ensure accuracy and uniformity over time. The relative abundances of bacterial taxa were then determined (Supplementary File S2).

### 2.4. Statistical Analyses

#### 2.4.1. α-Diversity

Primer_v7_ software (PRIMER-E Ltd., Ivybridge, UK) was used to generate Shannon and Simpson indices (within-subject comparisons) using normalized taxa relative abundance data [20,21]. Comparisons were performed across the sampling timeline of a four-week treatment period, at baseline (t = 0: group A) and during treatment at t = 1 wk (group B), t = 2 wk (group C), t = 3 wk (group D). We performed the following group comparisons: (i) A vs. BCD, (ii) AB vs. CD, (iii) A vs. B, (iv) A vs. C, and (v) A vs. D. Further, HPV+ vs. HPV− status comparisons were made for all timepoints combined and for the individual timepoints A, B, C, or D. Mann-Whitney U-tests were used to determine significance (α = 0.05) using XLSTAT_v2021_._2_._2_, with adjustment for the False Discovery Rate (FDR) [22].

#### 2.4.2. β-Diversity

PRIMER_v7_ (PRIMER-E Ltd., Ivybridge, UK) was used to perform longitudinal *β*-diversity (between subjects) comparisons between groups, as mentioned above [21]. Additionally, *β*-diversity was determined on cross-sectional comparisons for HPV+ vs. HPV− groups. Normalized abundance data were squared-root transformed and converted to Bray–Curtis similarity matrices. PERMANOVA analyses were completed using a mixed model with unrestricted permutation of raw data, 9999 permutations, and type III partial sum of squares, as previously implemented [11,17]. Fixed factors in the design consisted of ‘Timepoint’ for longitudinal comparisons and ‘HPV Status’ for cross-sectional comparisons. Monte-Carlo corrected *p*-values (α = 0.05) were determined due to their ability to accurately represent the probability of observing a result at least as extreme as what was observed in complex situations, such as oral microbial communities that can contain many bacterial taxa. In this pilot study, variables such as gender and age were not significantly different between groups (*p* < 0.05) and incorporating them in the PERMANOVA design would reduce statistical power in an already small sample size, when comparing 18 HPV(+) to 46 HPV(−) samples. Therefore, age and gender were ignored as possible covariates. Further, the R package ‘LongDat’ was utilized to determine longitudinal significance of the time series analysis with ‘Timepoint’ and ‘HPV status’ defined as covariates [23]. Microbiome Multivariable Association with Linear Models (MaAsLin2) was used to perform a multivariable association analysis where the univariate outcome (per-feature taxa) and the potentially multivariable metadata (Timepoint and HPV status) are modeled using linear and mixed model methods [24]. Additionally, Mann-Whitney U-tests were used to determine significance (α = 0.05) of taxa relative abundance differences when comparing HPV+ vs. HPV− groups.

#### 2.4.3. Linear Decomposition Model (LDM) Analysis

LDM_v6_._0_._1_ in R_v4_._4_._1_ was used to perform a linear decomposition analysis to compare the HPV+ group (n = 18 samples) to the HPV− group (n = 46 samples) across all timepoints (A–D), based on sequence reads. A principal component 2-D plot was generated based on Bray-Curtis dissimilarity distance after removing confounding effects from timepoint and gender. Further, LDM was run using Besag and Clifford stop rules, with a total of 15,000 permutations completed to determine whether HPV status was significantly associated with salivary microbiome (*p* < 0.05) and to identify significant taxa associated with HPV status (FDR-corrected *p*-adj < 0.10) [25]. PERMANOVA within LDM package comparing HPV+ to HPV− groups was performed using default 5000 maximum permutations. The LDM-A function within LDM package was called with the n.rarefy set to ‘all’ to determine association of HPV status with presence and absence of some bacteria (FDR = 0.1). Lastly, rarefaction curves were generated by plotting ‘Amplicon Sequence Variants’ (ASVs) obtained from the DADA2 pipeline in R_v4_._4_._1_ against ‘Sample Size’ by using the vegan_v2_._6–6_._1_ library.

#### 2.4.4. Receiver Operating Characteristic Curves

Receiver Operating Characteristic (ROC) curves were plotted for all species level taxa in common between MaAsLin2 analysis and FDR-corrected Mann-Whitney U-test using XLSTAT_v2021_._2_._2_. Species level taxa with an Area Under the Curve (AUC) >0.800 were subjected to Combinational ROC analysis using the R package ‘CombiROC’ to determine the strength of multi-marker signature [26].

## 3. Results

**Figure 1:**
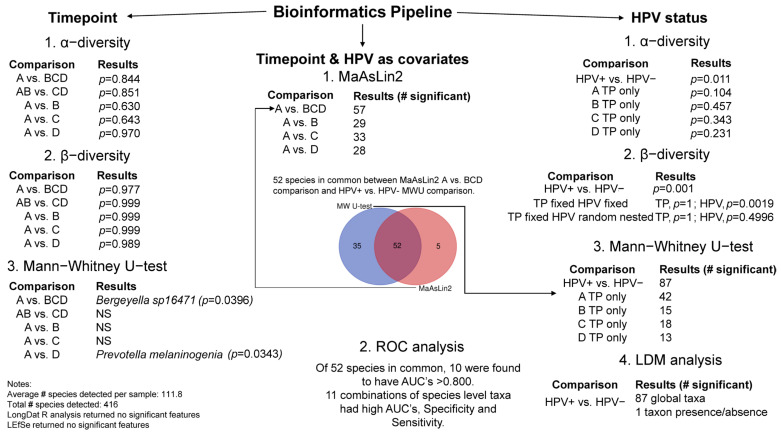
Overall analytical strategy. Legend. Overall analytical strategy of bioinformatic analysis for the following timepoint comparisons are shown: (i) A vs. BCD; (ii) AB vs. CD; (iii) A vs. B; (iv) A vs. C; and (v) A vs. D. Further, the following HPV status comparisons were analyzed: (i) HPV+ vs. HPV− All timepoints; (ii) HPV+ vs. HPV− A timepoint only; (iii) HPV+ vs. HPV− B timepoint only; (iv) HPV+ vs. HPV− C timepoint only; (v) HPV+ vs. HPV− D timepoint only. Primer_v7_ software was used to generate Shannon and Simpson indices (PRIMER-E Ltd., Ivybridge, UK) to compare *α*-diversities. Mann–Whitney U-tests were used to determine significance (a = 0.05) using XLSTAT_v2021_._2_._2_. PRIMER_v7_ was used to perform longitudinal and cross-sectional *β*-diversity comparisons. Normalized abundance data were squared-root transformed and converted to Bray–Curtis similarity matrices. PERMANOVA analyses was completed using a mixed model with unrestricted permutation of raw data, 9999 permutations, and type III partial sum of squares. Fixed factors in the design consisted of ‘Timepoint’ for longitudinal comparisons and ‘HPV Status’ for cross-sectional comparisons. Monte-Carlo corrected *p*-values (α = 0.05) were determined. The R package ‘LongDat’ was used to determine longitudinal significance of time series analysis with ‘Timepoint’ and ‘HPV status’ as covariates. Microbiome Multivariable Association with Linear Models (MaAsLin2) was used to perform a multivariable association. Mann–Whitney U-tests were used to determine significance (α = 0.05) of individual taxa relative abundance differences comparing the HPV+ group to HPV− group. There was a total of 52 species taxa in common regarding ‘HPV’ status between MaAsLin2 and Mann–Whitney U-test analyses. The Linear Decomposition Model (LDM_v6_._0_._1_) R_v4_._4_._1_ package was utilized on HPV+ vs. HPV− relative abundance data from all timepoints. The function LDM provides global tests of any effect of the microbiome and the specific tests. LDM was run removing confounders of ‘Gender’ and ‘Timepoint’ to see if HPV status was associated with the microbiome. Using Besag and Clifford stop rules, a total of 15,000 permutations were completed. After accounting for ‘Gender’ and ‘Timepoint’, ‘HPV status’ was significantly associated with the microbiome (LDM *p*-value = 0.003). Using FDR corrected *p*-values (*p*-adj < 0.10), we identified 87 taxa that contributed to the associated of HPV status. A total of 54 species taxa were identified to be in common between HPV+ vs. HPV− Mann-Whitney U-tests and LDM analysis. Receiver Operating Characteristic (ROC) curves were plotted for all species level taxa in common between MaAsLin2 analysis and Mann-Whitney U-test using XLSTAT_v2021_._2_._2_. Species level taxa with an Area Under the Curve (AUC) >0.800 were subjected to Combinational ROC analysis using the R package ‘CombiROC’ to determine the strength of the multi-marker signature.

Figure 1 shows the overall methodology with results of the bioinformatics analysis. Sample sizes of group comparisons with the number of detected taxa are shown in Table 1. Total number of taxa detected across all samples was n = 416.

### 3.1. α- and β-Diversity

No *α*-diversity comparisons were determined as significant using Timepoint as a variable. The only *α*-diversity comparison that was significant using HPV status was the comparison of HPV+ vs. HPV− including all sample timepoints (‘A’, ‘B’, ‘C’, and ‘D’; *p* = 0.011) and was marginally significant after FDR correction (p_adj_ = 0.055). Additionally, no longitudinal β-diversity comparisons were determined as significant using ‘Timepoint’ as the fixed factor in the PERMANOVA design within PRIMER_v7_. When analyzing the cross-sectional data (HPV+ vs. HPV− with all timepoints), PRIMER_v7_ determined the comparison to be significant (β-diversity; *p* = 0.001). Considering ‘Timepoint’ and ‘HPV’ as fixed variables within the cross-sectional analysis (HPV+ vs. HPV− with all timepoints), HPV status was returned as significant (β-diversity; *p* = 0.0019).

### 3.2. LDM Analysis

Based on the longitudinal analysis of a small population of samples, including 18 HPV(+) and 46 HPV(−) samples from HNC patients (N = 5 and N = 12, respectively, across all timepoints (A-D)), we found the effects on salivary microbiome from durvalumab treatment to be negligible. Since LDM provides global tests of any effect of the microbiome, LDM was further run by removing confounders of Gender and Timepoint to determine whether HPV status was associated with the microbiome. Based on Besag and Clifford stop rules, a total of 15,000 permutations were completed [25]. After accounting for gender and timepoint, HPV status was significantly associated with the microbiome (LDM *p*-value = 0.003). Using FDR corrected *p*-values (*p*-adj < 0.1) we identified 87 bacterial species that contributed to association with HPV status (Table 2). Based on relative abundance, 84 species were overrepresented in HPV(+) samples, including the Gram-positive species *Corynebacterium durum* and *Gemella sanguinis*, and the Gram-negative species *Veillonella atypica*. *Prevotella melaninogenica* was determined as having the largest population level mean relative abundance at 0.0475; *Lactobacillus gasseri* and *Veillonella atypica* followed with population level mean relative abundances of 0.0286 and 0.0252, respectively. Additionally, two other *Lactobacillus* species were revealed to be overrepresented in HPV(−) samples (Table 2).

**Figure 2:**
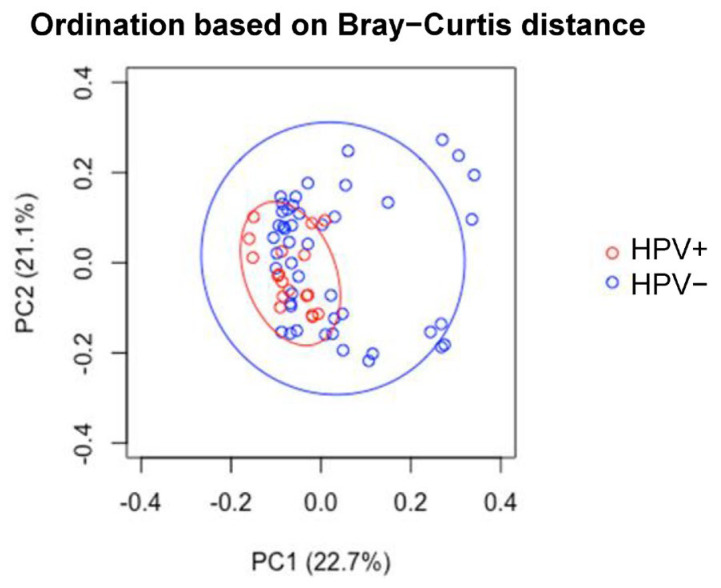
Decomposition model PCA plot output. Legend. PCA plot showing the performance of two distance-based ordination principal components, based on the Bray-Curtis distance of HPV status after removing the confounding effects from timepoint and gender. The ellipses are at 90% confidence limits.

**Figure 3:**
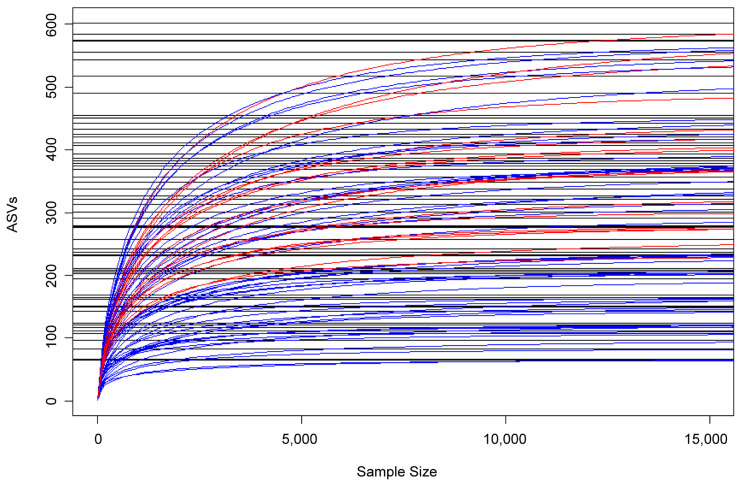
HPV+ HPV− rarefaction curves. Legend. Using the vegan_v2_._6–6_._1_ library, rarefaction curves were generated using Amplicon Sequence Variants (ASVs) data across all timepoints (A–D) from the DADA2 pipeline in R_v4_._4_._1_. The *x*-axis represents sample size and the *y*-axis shows the number of ASVs plotted using HPV+ (N = 18, red) and HPV− (N = 46, blue) data.

**Figure 4:**
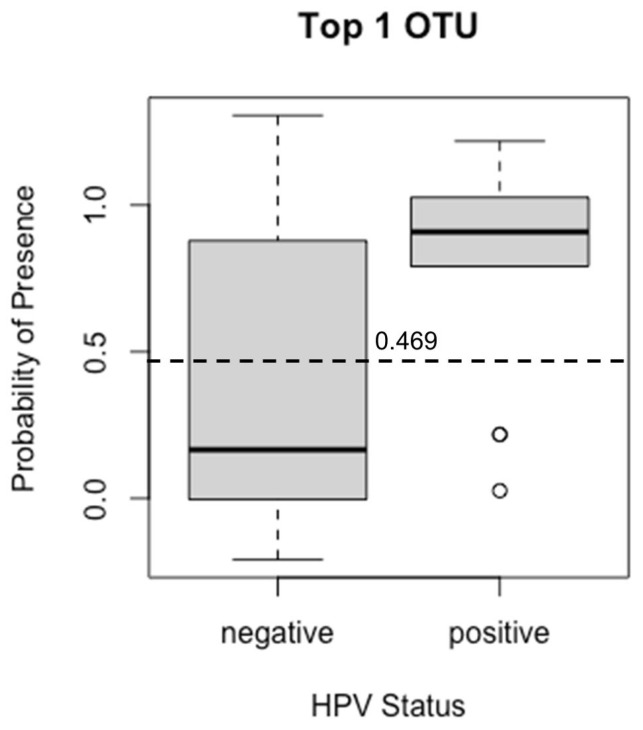
Plot showing probability of presence of *Veillonella atypica*. Legend. Within LDM-A, the LDM function with the n.rarefy set to ‘all’ was called. This returned a global *p*-value of 0.04866667 showing HPV status is marginally associated with the presence and absence of some bacteria. At an FDR of 0.1, we identified that *Veillonella atypica* was differentially present among HPV+ and HPV− patients (raw *p*-value = 0.000255, adjusted *p*-value = 0.0869, population-averaged probability of presence = 0.469, direction of covariate effect in relation to HPV− group = Negative).

In addition, LDM principal component analysis (PCA) analysis showed clustering of HPV(+) samples, whereas HPV(−) samples were scattered (Figure 2), consistent with corresponding rarefactions curves (Figure 3). Moreover, in relation to HPV status, PERMANOVA analysis using taxa relative abundances within LDM package (5000 permutations) returned an R-squared value of 0.0518 (the proportion of distance variance explained by each trait), after projecting off Gender and Timepoint, confirming association (*p* = 0.038). At last, by using the LDM-A presence/absence function, *Veillonella atypica* was found to be the top OTU significantly more present in HPV+ group (*p*-adj < 0.1), with a probability of presence of 0.469, in agreement with findings in our previous study on HPV+ and HPV− head and neck cancer patients [11] (Figure 4).

### 3.3. Mann–Whitney U-Tests

Mann-Whitney U-test results for the ‘Timepoint’ comparison resulted in *Bergeyella sp16471* being significant in the A vs. BCD group (FDR corrected *p* = 0.0396) and overrepresented among the BCD group. Further, *Prevotella melaninogenica* was found significant in A vs. D groups (FDR corrected *p* = 0.0343) and overrepresented in D group. Results for the HPV status resulted in 87 significant species taxa for the HPV+ vs. HPV−, 42 significant species taxa for the A timepoint only, 15 significant species taxa for the B timepoint only, 18 significant species taxa for the C timepoint only, and 13 significant species taxa for the D timepoint only. Table 2 shows bacterial species commonly significant between LDM analysis and Mann–Whitney U-test. Species taxa in common included eight *Actinomyces* species, eight *Streptococcus* species, and three *Prevotella* species. All eight *Actinomyces* and *Streptococcus* taxa were associated with HPV(+) status, while one of the three *Prevotella* species was associated with HPV(−) status.

### 3.4. MaAsLin2 and ROC Analysis

**Figure 5:**
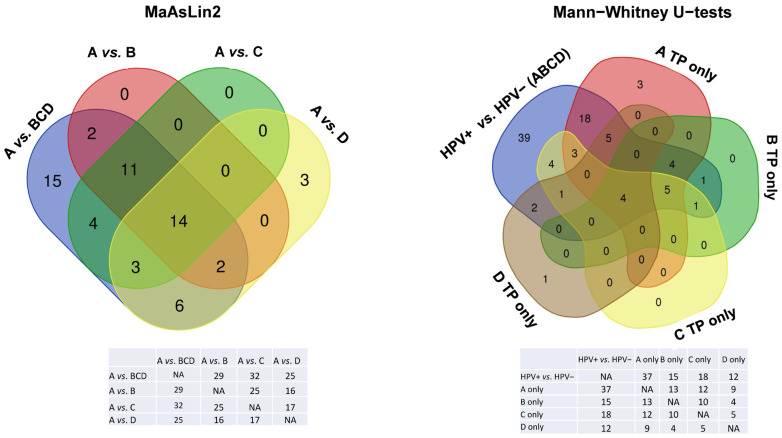
Venn diagrams of species in common among the MaAsLin2 and Mann-Whitney U-test results. Legend. Species taxa in common between longitudinal comparisons using Multivariable Association with Linear Models (MaAsLin2) and Mann-Whitney U-tests of taxa relative abundance differences comparing the HPV+ group to the HPV− group. Tables below each comparison summarize the Venn diagrams.

**Figure 6:**
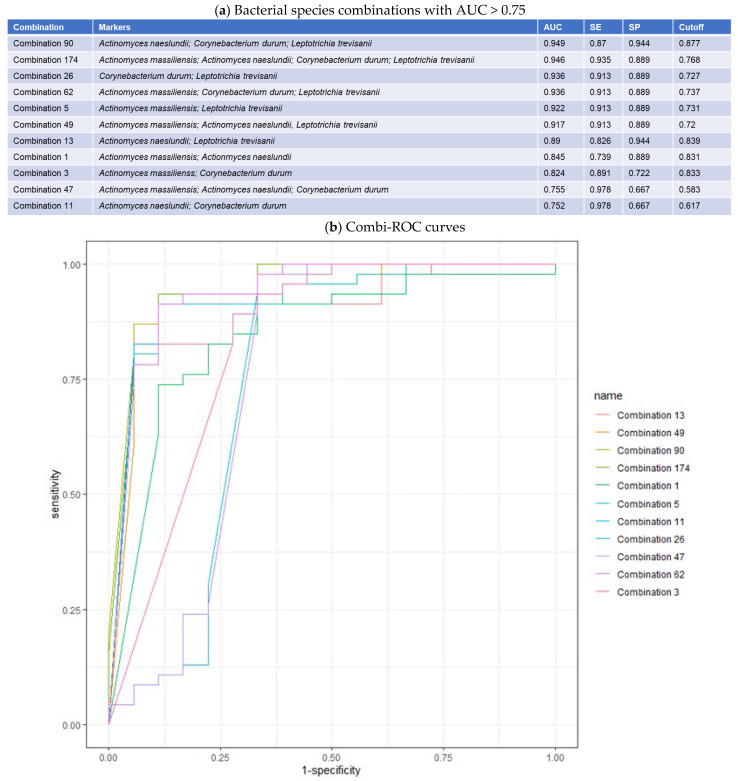
Multi-ROC analysis of significant taxa overrepresented in HPV+ patients. Legend. (**a**) A total of 11 combinations represented by 2–4 bacterial species among 10 possible designated as ‘Markers’ showing the Area Under the Curve (AUC), specificity (SP), sensitivity (SE), and detection threshold (Cutoff) are shown. (**b**) Graphical representation of CombiROC curves.

MaAsLin2 analysis resulted in the A vs. BCD group comparison having 57 significant taxa (Table 3), A vs. B having 29 significant species, A vs. C having 33 significant species, A vs. D having 28 significant species. Figure 5 shows Venn diagrams of the species in common among the comparisons. Total of 52 species were in common between MaAsLin2 A vs. BCD group comparison and the HPV+ vs. HPV− Mann–Whitney U-test comparison. ROC curves of the 52 species in common resulted in 10 being significant with AUC’s >0.800. Combinational ROC curves resulted in 11 combinations of species-level taxa having high AUC, specificity, and sensitivity, shown in Figure 6a. Species taxa representative of the combinational ROC plot (Figure 6b) and their respective fold changes are presented in Table 4.

## 4. Discussion

This longitudinal study showed for the first time that PD-L1 inhibitor durvalumab immunotherapy has a negligible effect on saliva oral microbiome composition overall. When investigating the community of 416 species taxa detected, HPV status accounted for most of differences in saliva oral microbiome composition. However, the small sample size in this study significantly reduces statistical power, negatively affecting the likelihood of identifying true effects in this study and, therefore, results require validation using a larger cohort over a greater span of time.

Nevertheless, LDM showed distinct clustering of HPV+ and HPV− samples. LDM indicated that HPV status is significantly associated with the salivary microbiome when removing gender and timepoint as variables. LDM identified 84 species taxa which were associated with HPV+, including taxa belonging to families previously implicated for their involvement. In a study by Pavlova et al., they reported that *Streptoccus* promotes HPV infection into oral squamous cell carcinoma cells [27]. We were able to show eight *Streptococcus* spp. and eight *Actinomyces* spp. associated with HPV(+) status via LDM analysis overlapping with Mann–Whitney U-test significant species, contributing to beta-diversity differences (Table 2). *Veillonella atypica* was found in agreement with findings in our previous study of a larger cohort of patients on HPV+ (N = 16 patients; n = 64 samples) and HPV− (N = 7 patients; n = 28 samples) head and neck cancer patients prior to any cancer treatment including saliva, buccal mucosa, superficial subgingival plaque, and tongue samples [11]. *Veillonella atypica* is associated with a higher relative abundance in HPV+ individuals.

In addition, two previous studies identified the taxa families *Actinomycetaceae*, *Prevotellaceae*, *Veillonellaceae*, *Campylobacteraceae*, and phylum Bacteroidetes to be more abundant in oral samples of HPV+ individuals, and *Gemellaceae* to be less abundant [28,29]. In this pilot study, when considering the taxa *Actinomycetaceae*, *Prevotellaceae*, *Campylobacteraceae*, and Bacteroidetes, we observed similar trends of higher abundances in HPV+ samples. Among significant *Veillonellaceae*, we found 50% of significant species to have higher abundance in HPV+ samples. Among significant *Gemella* species taxa, we found one species to have higher abundance in HPV+ samples (*G. sanguinis*) while the other had lower abundance in HPV+ samples (*G. haemolysans*). *G. haemolysans* has been shown to inhibit the growth of *Porphyromonas gingivalis* in a study investigating the progression of periodontitis and should be explored as a species with possible protective effects on the oral microbiome [30].

Based on Mann–Whitney U-tests on timepoint data, we also found *Bergeyella sp16471* to be overrepresented in the ‘BCD’ timepoints (*p* = 0.0396) in HPV+ patients. A previous study investigating the oral microbiome before and after chemotherapy suggested that *Bergeyella* may have a protective effect on dysbiosis and oral mucositis in pediatric patients diagnosed with solid or hematological malignancy [31]. The relationship between *Bergeyella* in HNC patients undergoing immunotherapy should be explored further using a larger sample size. Moreover, in timepoint D, *P. melaninogenica* was overrepresented when comparing A vs. D (*p* = 0.0343). Per our LDM analysis, *P. melaninogenica* was found to significantly contribute to HPV+ status (*p* = 0.00377). *P. melaninogenica* has been previously implicated as a pathogenic species contributing to periodontitis and OSCC. *P. melaninogenica* was one of 52 overlapping species taxa from LDM analysis that overlapped with Mann–Whitney U-tests comparing HPV+ vs. HPV− samples across all timepoints (Table 2). Overlapping species taxa also included *G. sanguinis*, *Corynebacterium durum*, and *V. atypica*.

MaAsLin2 analysis identified 57 significant bacterial species when comparing the A timepoint to all others (B, C, and D) (Table 3). Of the 57 species, 52 were in common between MaAsLin2 and the Mann–Whitney U-test significant species taxa when comparing HPV+ samples to HPV− samples including *G. sanguinis*, *C. durum*, and *V. atypica* (Table 3, Figure 5).

*Actinomyces sp4769* was determined to be the most significant in MaAsLin2, second most significant in LDM analysis, and had the highest AUC at 0.932, although it was not part of any CombiROC panel of bacterial species. *Actinomyces* genus has been linked to oral cavity cancers [7,32,33]. Actinomycosis, a chronic infection of *Actinomyces* in the oral cavity was shown to resemble head and neck carcinomas [34].

When comparing the results of this study to our previous study investigating the oral microbiome profiles of oral samples (buccal mucosa, saliva, superficial subgingival plaque, and tongue) of 30 patients diagnosed as HPV+ or HPV− undergoing radiation treatment for head and neck cancer, we found species/genera in common. *G. sanguinis* was thus previously identified as a significant feature in the adjusted [log(RA + 1)] combinational ROC curve [11].

In the current study, although *G. sanguinis* did not constitute a part of the top 11 combinational ROC curves, it was present in the top 10 individual ROC curves that were used as input for CombiROC, having a high individual AUC of 0.903. In addition, *C. durum* was notably present in seven of 11 (63.6%) combinational ROC curves (Figure 6), although in our previous study, this species was identified as a differential taxon of HPV− patients in a comparison that included patients who received antibiotics within two weeks of sampling [11]. Further, we previously determined that *Veillonella* spp. were part of a group that represented distinct taxa for HPV+ samples in HNC patients, confirming results of other studies [9,11,35,36].

Additionally, we found the genera *Leptotrichia* to be in common between the two studies, both showing the relative abundance of *Leptotrichia* spp. to be higher in oral samples of HPV+ patients, despite the differences in sample sizes between HPV+ and HPV− groups. Within this study, *Leptotrichia* spp. overlapped between the LDM analysis and Mann–Whitney U-tests comparing HPV groups. Furthermore, *Leptotrichia* spp. were part of the combinational ROC curves in our previous study, as well as being present in seven of 11 (63.6%) of combinational ROC curves in this study [11]. One *Leptotrichia* species (*L. amnionii*) was previously associated with HPV infection and with increased stage of cervical cancer carcinogenesis in a study by Nawrot et al. [16].

Multiple studies have evaluated the potential for saliva to serve as a diagnostic tool in diseases such as head and neck cancer, diabetes, neurodegenerative diseases, and immunodeficiencies [37,38]. Currently, salivary diagnostic test strips are available to test for *Streptococcus mutans* and lactobacilli to determine dental caries risk [39]. Here, we suggest there is a potential to utilize the bacterial species identified in this study, using high AUC, specificity, and sensitivity as non-invasive salivary diagnostic markers to detect HPV and assess possible HNC risk (Table 4).

*Veillonella atypica* is rarely described as an opportunistic bacterial species. It is known as an early colonizer, forming biofilms with *Streptococcus* and/or *Porphyromonas* species [40]. These species taxa were identified as significantly associated with HPV+ status in this study and have previously been implicated as predictive markers for the oral lesion-OSCC transition, suggesting presence of the three taxa together might represent an indicator of HPV infection in HNC patients [41] (Table 2). Additionally, *G. sanguinis*, has been described as an opportunistic species causing infective endocarditis in rare instances in immunocompromised individuals, such as those diagnosed with OPCs [42,43,44]. Further, *C. durum* has been shown to protect species from the innate immune response, such as the caries and periodontal disease associated species *Streptococcus sanguinis* [45]. Although the relationship between these species and HPV infection in HNCs is still unclear, by blocking programmed cell death ligand-1 and enhancing the function of T cells, durvalumab may guard against infection-causing species while having minimal effects on the overall salivary microbial composition, representing more than 700 bacterial species [46].

While our findings may essentially have descriptive value, a larger sample size with a balanced design and a longer sampling timeline is suggested for confirmation as well as to determine whether these data are specific to durvalumab compared to alternative immunotherapy. In particular, the small sample size of HPV+ patients in this study is a significant limiting factor that may affect the robustness of our results. Further, there are other factors that were not considered in this study that may influence the oral microbiome composition, including diet, pre-existing conditions, and prescription medications [47,48,49].

## 5. Conclusions

This study suggests that durvalumab immunotherapy has negligible effects on the overall composition of the salivary microbiome; however, one may not preclude possible impact on opportunistic oral pathogens in the context of HPV status. Future directions of this study include analysis of HNC HPV patients treated with alternative immunotherapies compared to healthy controls, with the consideration of possible confounders such as diet and comorbidities.

## Figures and Tables

**Table 1:** Sample sizes of comparisons and detection of bacterial taxa.

Timepoint Comparisons	Samples	Taxa	Samples	Taxa
	A		BCD	
A vs. BCD	17	373	47	391
	AB		CD	
AB vs. CD	34	400	30	370
	A		B	
A vs. B	17	373	17	361
	A		C	
A vs. C	17	373	17	347
	A		D	
A vs. D	13	373	13	336
**HPV comparisons**	**HPV+ samples**	**Taxa**	**HPV− samples**	**Taxa**
HPV+ vs. HPV− all timepoints	18	299	46	376
HPV+ vs. HPV− A only	5	281	12	326
HPV+ vs. HPV− B only	5	234	12	335
HPV+ vs. HPV− C only	5	247	12	317
HPV+ vs. HPV− D only	3	217	10	314

Comparisons consisted of longitudinal analyses prior to immunotherapy (A) and during immunotherapy sampled one week apart (B = 1 week; C = 2 weeks; D = 3 weeks) and cross-sectional analysis of HPV positive (HPV+) patients compared to HPV negative (HPV−) patients. Number of samples included in the comparison and bacterial taxa detected are shown.

**Table 2:** Bacterial species contributing to HPV status per LDM analysis.

Taxa ^a^	Raw *p*-Value ^b^	Adjusted *p*-Value ^c^	Population Level Mean RA ^d^	Direction ^e^
***Actinomyces massiliensis***	8.33 × 10^−5^	0.00251	0.000105	-
***Actinomyces sp4769***	8.33 × 10^−5^	0.00251	0.000363	-
***Corynebacterium durum***	8.33 × 10^−5^	0.00251	0.000305	-
***Gemella sanguinis***	8.33 × 10^−5^	0.00251	0.0059	-
***NA sp31630***	8.33 × 10^−5^	0.00251	0.000742	-
***Oribacterium sinus***	8.33 × 10^−5^	0.00251	0.00664	-
***NA sp37035***	8.33 × 10^−5^	0.00251	0.00196	-
***Veillonella atypica***	8.33 × 10^−5^	0.00251	0.0252	-
***Leptotrichia trevisanii***	8.33 × 10^−5^	0.00251	0.000311	-
***Actinomyces naeslundii***	0.000167	0.00377	0.000376	-
***Prevotella melaninogenica***	0.000167	0.00377	0.0475	-
***Streptococus dentisani mitis***	0.000167	0.00377	0.00205	-
***Selenomonas noxia***	0.000333	0.00696	0.00114	-
***Streptococcus australis***	0.000417	0.00754	0.000792	-
***Streptococcus constellatus intermedius***	0.000417	0.00754	0.000797	-
***Selenomons p37072***	0.000583	0.00989	0.000208	-
***Gardnerella vaginalis***	0.000917	0.0131	0.000172	-
***Howardella sp32242***	0.000917	0.0131	0.000299	+
***NA sp35322***	0.000917	0.0131	0.00116	-
***Corynebacterium matruchotii***	0.0015	0.0194	0.000799	-
***Rothia aeria***	0.0015	0.0194	0.000266	-
*Streptococcus infantis parasanguinis*	0.00175	0.0216	0.000434	-
***Atopobium parvulum***	0.00208	0.0246	0.00835	-
*Alloprevotella sp13512 sp13518*	0.0025	0.0258	0.000126	-
*NA sp31584*	0.0025	0.0258	1.42 × 10^−5^	-
***Leptotrichia hofstadii***	0.0025	0.0258	7.64 × 10^−5^	-
***NA sp4820***	0.00258	0.0258	0.000131	-
*Prevotella scopos*	0.00267	0.0258	0.000237	-
***NA sp35348***	0.00283	0.0261	0.000904	-
*Alloprevotella sp13517*	0.00308	0.0261	0.000138	-
*Lactobacillus iners*	0.00308	0.0261	3.94 × 10^−5^	-
***Streptococcus infantis sanguinis***	0.00308	0.0261	0.000365	-
*Peptoclostridium sp34351*	0.00342	0.0281	1.12 × 10^−5^	-
***Prevotella nanceiensis***	0.00383	0.0297	0.000806	-
*Neisseria sp49954*	0.00383	0.0297	0.000181	-
***Streptococcus cristatus***	0.00425	0.032	0.00611	-
*Leptotrichia buccalis hofstadii*	0.0045	0.0322	2.98 × 10^−5^	-
*Porphyromonas gingivalis*	0.00458	0.0322	0.000156	-
***NA sp31682***	0.00475	0.0322	0.000332	-
*NA sp37532*	0.00475	0.0322	0.000675	-
***Streptococcus lactarius parasanguinis***	0.005	0.0331	0.000369	-
***Porphyromonas endodontalis***	0.00525	0.0339	0.000799	-
***Lachnoanaerobaculum orale***	0.00567	0.0349	0.0031	-
***Leptotrichia sp37519***	0.00567	0.0349	0.00654	-
*Bacteroides sp12340*	0.006	0.0354	0.000204	-
***NA sp66013***	0.006	0.0354	0.00328	-
***Streptococcus sp30088***	0.00617	0.0355	0.000304	-
***Lactobacillus fermentum***	0.00642	0.0355	0.0132	+
***NA sp19816***	0.0065	0.0355	6.1 × 10^−5^	-
***Stomatobaculum longum***	0.00658	0.0355	0.00447	-
*NA sp32067*	0.00667	0.0355	8.71 × 10^−5^	-
*Selenomonas sp37074*	0.0075	0.0391	0.000275	-
***Mogibacterium vescum***	0.00825	0.0422	0.00321	-
***Streptococcus intermedius***	0.00875	0.044	0.00114	-
***Actinomyces johnsonii***	0.00925	0.0449	0.000187	-
***Actinomyces gerencseriae***	0.00927	0.0449	0.000272	-
*Neisseria sicca*	0.00973	0.0463	2.06 × 10^−5^	-
***Fusobacterium periodonticum***	0.0111	0.0516	0.00707	-
***Scardovia wiggsiae***	0.0112	0.0516	0.000256	-
*Moryella sp33027*	0.0118	0.0533	8.17 × 10^−6^	-
*Desulfovibrio sp52730*	0.0136	0.0606	7.93 × 10^−6^	-
*Streptococcus infantis oralis sanguinis*	0.0144	0.0629	0.000125	-
***Lactobacillus gasseri***	0.0153	0.0654	0.0286	+
*Streptococcus sobrinus*	0.0154	0.0654	3.92 × 10^−5^	-
***Actinomyces oris***	0.0168	0.0703	0.00258	-
***Campylobacter concisus***	0.0174	0.0712	0.00288	-
*Kocuria kristinae*	0.0177	0.0712	2.71 × 10^−5^	-
***Porphyromonas sp13376***	0.0178	0.0712	0.00434	-
*Neisseria flavescens mucosa*	0.0185	0.0717	0.000108	-
*NA sp66034*	0.0185	0.0717	5.73 × 10^−5^	-
*Streptococcus infantis oralis parasanguinis*	0.0188	0.072	2.62 × 10^−5^	-
***Actinomyces sp4767***	0.0204	0.0766	7.17 × 10^−5^	-
*Lachnoanaerobaculum umeaense*	0.0206	0.0766	0.000172	-
*NA sp13878*	0.0214	0.0785	0.000492	-
***Prevotella dentalis***	0.022	0.0796	0.000268	-
*Mogibacterium neglectum vescum*	0.0236	0.0843	5.2 × 10^−5^	-
***Actinomyces dentalis orihominis***	0.025	0.0861	0.000735	-
*Lacobacillus crispatus*	0.025	0.0861	4.82 × 10^−5^	-
*Prevotella jejuni melaninogenica*	0.0254	0.0861	1.94 × 10^−6^	-
*NA sp32913*	0.0254	0.0861	2.09 × 10^−6^	-
*Selenomonas artemidis*	0.026	0.0871	0.000533	-
***Alloscardovia omnicolens***	0.0265	0.0877	0.00125	-
***Lactobacillus rhamnosus***	0.0275	0.0896	0.00137	+
*NA sp31126*	0.0278	0.0896	8.06 × 10^−5^	-
***Prevotella pleuritidis***	0.0285	0.091	0.000359	+
*Musa textilis*	0.031	0.0975	3.09 × 10^−6^	-
*Actionmyces dentalis orihominis sp4753*	0.0312	0.0975	0.000164	-

^a^ Taxa that contribute to HPV status. ^b^ Linear Decomposition Model (LDM) raw *p*-value prior to correction consisting of 15,000 permutations using the stopping rule of Besag and Clifford. ^c^ LDM FDR adjusted *p*-value. ^d^ Population level mean relative abundance. ^e^ Direction of the covariate effect in relation to HPV− status, with minus (-) meaning that HPV− status is associated with lower RA than the HPV+ status, and plus (+) signifying that the HPV− status is associated with higher RA than HPV+ status. Note: Species taxa shown in **bold** are commonly significant with Mann–Whitney U-test (n = 54).

**Table 3:** MaAsLin2 significant taxa A vs. BCD.

Feature ^a^	Effect Size ^b^	Standard Error ^c^	*p*-Value ^d^
***Actinomyces sp4769***	3.650674	0.313348	6.19 × 10^−17^
***Corynebacterium durum***	2.671046	0.37376	1.53 × 10^−9^
***NA sp37035***	4.475144	0.618709	1.09 × 10^−9^
***Leptotrichia trevisanii***	2.324937	0.366879	3.54 × 10^−8^
***Actinomyces massiliensis***	1.709514	0.299844	4.03 × 10^−7^
***Oribacterium sinus***	2.070299	0.369039	5.68 × 10^−7^
***Gemella sanguinis***	4.292765	0.820719	2.34 × 10^−6^
***Actinomyces naeslundii***	2.28903	0.46307	6.70 × 10^−6^
***NA sp31630***	2.821748	0.570336	6.59 × 10^−6^
***Streptococcus australis***	2.233489	0.512714	5.36 × 10^−5^
***Selenomonas noxia***	2.720517	0.627544	5.76 × 10^−5^
***Gardnerella vaginalis***	1.471213	0.351596	9.64 × 10^−5^
***Porphyromonas endodontalis***	2.49114	0.59271	9.05 × 10^−5^
*Streptococcus dentisani mitis*	3.326154	0.78488	8.04 × 10^−5^
***Howardella sp32242***	−2.15071	0.510985	8.87 × 10^−5^
***NA sp35348***	2.716136	0.682613	0.000192
***Leptotrichia sp37519***	3.237624	0.818936	0.000209
***Rothia aeria***	1.271655	0.325619	0.000245
*Streptococcus constellatus intermedius*	2.421821	0.630378	0.000301
***Selenomonas sp37072***	1.689886	0.44746	0.000372
***Leptotrichia wadei***	3.302522	0.876053	0.00038
***Streptococcus cristatus***	2.164533	0.578395	0.000415
***Veillonella atypica***	3.225777	0.864835	0.000432
*Streptococcus infantis sanguinis*	1.654843	0.445433	0.000453
***Lactobacillus fermentum***	−3.49841	0.968022	0.000625
***Actinomyces gerencseriae***	1.78548	0.509526	0.000881
***Actinomyces oris***	2.590536	0.737814	0.000862
***Corynebacterium matruchotii***	1.831948	0.545537	0.001379
***Actinomyces johnsonii***	1.35515	0.408988	0.001578
***Scardovia wiggsiae***	1.531826	0.464295	0.001647
***Haemophilus parainfluenzae***	2.849527	0.877864	0.001931
***Stomatobaculum longum***	1.97897	0.625083	0.002446
*Actinomyces dentalis orihominis*	1.923268	0.613446	0.002677
***Streptococcus infantis***	2.39051	0.763754	0.002718
***NA sp4820***	1.060871	0.342166	0.002961
***NA sp31682***	1.59527	0.515906	0.003033
***Leptotrichia hofstadii***	0.882742	0.284776	0.002967
***Lachnoanaerobaculum orale***	1.992016	0.649435	0.003259
***Streptococcus intermedius***	1.966111	0.646455	0.003511
***NA sp37033***	1.753744	0.576544	0.003506
***Streptococcus sp30088***	0.995509	0.329461	0.003714
***NA sp35322***	2.10789	0.697398	0.003705
***Alloscardovia omnicolens***	2.002643	0.667595	0.003953
***Atopobium parvulum***	1.44814	0.491433	0.004592
***bacterium enrichment***	1.77799	0.602781	0.004555
*Streptococcus lactarius parasanguinis*	1.392599	0.472742	0.004605
***Prevotella denticola***	1.967924	0.674051	0.004957
***Campylobacter concisus***	1.702208	0.58613	0.005175
***Rothia dentocariosa***	1.973771	0.681746	0.005306
***Lactobacillus gasseri***	−3.21474	1.141644	0.006604
***Streptococcus gordonii***	2.114021	0.765832	0.00768
***Prevotella buccae***	−0.8825	0.321439	0.007996
***NA sp19816***	0.830921	0.308142	0.009117
***Prevotella dentalis***	1.337058	0.513677	0.011672
***Pseudoramibacter alactolyticus***	−1.22081	0.470152	0.011866
***Fusobacterium periodonticum***	2.455544	0.9578	0.012923
***Veillonella sp37198***	−0.96167	0.376411	0.013224

Legend. ^a^ Significant taxa (n = 57) using the Microbiome Multivariable Association with Linear Models (MaAsLin2) package within R comparing timepoint A with timepoints B, C, D (A vs. BCD). ^b^ The model coefficient value. ^c^ The standard error from the model. ^d^ Benjamini–Hochberg corrected *p*-value (*p* < 0.05). Taxa shown in bold correspond to those in common between MaAsLin2 A vs. BCD and Mann-Whitney U-test HPV+ vs. HPV− comparisons (n = 52).

**Table 4:** Fold changes of the 10 species with AUC’s > 0.800 from MaAsLin2 longitudinal and Mann-Whitney U-test cross-sectional analysis resulting in 52 significant species in common.

Species with AUC > 0.800 (n = 10) ^a^	*p*-Value ^b^	AUC ^c^	Threshold ^d^	SE ^e^	SP ^f^	A ^g^	BCD_avg_^h^	FC ^i^	BCD Increase or Decrease ^j^	HPV− ^k^	HPV+ ^l^	FC ^m^	HPV+ Higher or Lower ^n^
*Actinomyces sp4769*	<0.0001	0.932	0.000327	0.833	1	0.0044	0.0063	0.4201	Increase	0.0009	0.0223	23.8868	Higher
*NA sp37035*	<0.0001	0.908	0.001	1	0.826	0.0490	0.0254	−0.4828	Decrease	0.0363	0.0888	1.4429	Higher
*Gemella sanguinis*	<0.0001	0.903	0.003522	1	0.696	0.0940	0.0812	−0.1361	Decrease	0.1458	0.2300	0.5776	Higher
*Oribacterium sinus*	<0.0001	0.89	0.007	0.778	0.87	0.1442	0.0931	−0.3549	Decrease	0.1791	0.2444	0.3645	Higher
***Leptotrichia trevisanii***	**<0.0001**	**0.837**	**0**	**0.778**	**0.87**	**0.0048**	**0.0050**	**0.0492**	**Increase**	**0.0019**	**0.0179**	**8.2892**	**Higher**
***Actinomyces naeslundii***	**<0.0001**	**0.831**	**0.00017**	**0.889**	**0.761**	**0.0043**	**0.0066**	**0.5226**	**Increase**	**0.0118**	**0.0121**	**0.0247**	**Higher**
*Veillonella atypica*	<0.0001	0.831	0.02	1	0.652	0.3840	0.4079	0.0622	Increase	0.8490	0.7587	−0.1063	Lower
*NA sp31630*	<0.0001	0.818	0.00019	0.889	0.696	0.0128	0.0115	−0.0979	Decrease	0.0157	0.0316	1.0059	Higher
***Corynebacterium durum***	**<0.0001**	**0.813**	**0.000196**	**0.667**	**0.957**	**0.0037**	**0.0053**	**0.4249**	**Increase**	**0.0017**	**0.0178**	**9.3332**	**Higher**
***Actinomyces massiliensis***	**<0.0001**	**0.812**	**0**	**0.722**	**0.891**	**0.0015**	**0.0017**	**0.1555**	**Increase**	**0.0017**	**0.0050**	**1.9731**	**Higher**

^a^ Species (n = 10) were determined from the 52 in common between the MaAsLin2 analysis on 416 species in the longitudinal comparison (A vs. BCD) and Mann-Whitney U-tests of 416 species in the cross-sectional comparison (HPV+ vs. HPV−, all timepoints). ^b^ *p*-value of Receiver Operating Characteristic (ROC) curve ^c^ Area Under the Curve (AUC). ^d^ Detection cutoff. ^e^ Sensitivity. ^f^ Specificity. ^g^ Sum of relative abundance for A timepoint. ^h^ Average sums of the B, C, and D timepoints. ^i^ Fold change of A timepoint vs. BCD timepoints. ^j^ Depiction of whether species taxa increases or decreases over time. ^k^ Sum of relative abundance for the HPV negative (HPV−) group. ^l^ Sum of relative abundance for the HPV positive (HPV+) group. ^m^ Fold change of the HPV+ group vs. HPV− group. ^n^ Depiction of whether species taxa were higher or lower in the HPV+ group. Species taxa shown in bold constituted the 11 combinational ROC curves.

## Data Availability

The datasets generated and analyzed are available from the corresponding author upon request. Supplementary Materials are available from GitHub at https://github.com/mbeckm01/HPV_OSCC (accessed on 20 December 2024).

## Supplementary Materials

The following supporting information can be downloaded at https://www.mdpi.com/article/10.3390/cancers17030452/s1, Supplementary File S1: DADA2 & Uclust parameters, Supplementary File S2: Sample Data.
